# Sonographic follow-up of diaphragm function in COVID-19: an exploratory study

**DOI:** 10.1183/23120541.00623-2022

**Published:** 2023-05-02

**Authors:** Carlijn Veldman, Wytze S. de Boer, Huib A.M. Kerstjens, Mireille A. Edens, Jan Willem K. van den Berg

**Affiliations:** 1 Department of Pulmonary Medicine, Isala Hospital, Zwolle, The Netherlands; 2 Department of Pulmonology, University of Groningen, University Medical Center Groningen, And Groningen Research Institute for Asthma and COPD GRIAC, Groningen, The Netherlands; 3 Epidemiology Unit, Department of Innovation and Science, Isala Hospital, Zwolle, The Netherlands; 4 C. Veldman and W.S. de Boer contributed equally

## Abstract

**Introduction:**

Survivors of COVID-19 frequently endure chronic disabilities. We hypothesise that diaphragm function has a long recovery time after COVID-19 hospitalisation and may play a role in post-COVID-19 syndrome. The aim of this study was to assess diaphragm function during COVID-19 hospitalisation and during recovery.

**Methods:**

We conducted a prospective single-centre cohort study in 49 enrolled patients, of which 28 completed 1-year follow-up. Participants were evaluated for diaphragm function. Diaphragm function was assessed using ultrasound measuring of diaphragm thickening fraction (TF) within 24 h after admission, after 7 days of admission or at discharge, whichever came first, and 3 and 12 months after hospital admission.

**Results:**

Estimated mean TF increased from 0.56 (95% CI 0.46–0.66) on admission to 0.78 (95% CI 0.65–0.89) at discharge or 7 days after admission, to 1.05 (95% CI 0.83–1.26) 3 months after admission and to 1.54 (95% CI 1.31–1.76) 12 months after admission. The improvements from admission to discharge, 3 months and 12 months were all significant (linear mixed modelling; p=0.020, p<0.001 and p<0.001, respectively), and the improvement from discharge to 3-month follow-up was borderline significant (p<0.1).

**Conclusion:**

Diaphragm function was impaired during hospitalisation for COVID-19. During recovery in hospital and up to 1-year follow-up, diaphragm TF improved, suggesting a long recovery time of the diaphragm. Diaphragm ultrasound may be a valuable modality in the screening and follow-up of (post-)COVID-19 patients for diaphragm dysfunction.

## Abstract 2

**This novel study challenges current investigative practices for patients with COVID-19. Future studies may be directed at the causative effects of diaphragm dysfunction and treatment effect of diaphragm training on persisting symptoms after COVID-19.** https://bit.ly/3ZeD3eo

## Introduction

Since the appearance of COVID-19, several studies have described chronic disabilities after recovery from infection with the SARS-CoV-2 virus. Various unresolved symptoms such as shortness of breath, cough or fatigue have been reported in COVID-19 survivors several months after hospital discharge, sometimes referred to as long COVID-19 or post-COVID-19 syndrome [1–3]. Although it is not fully understood why these symptoms occur, neuromuscular involvement including the diaphragm has been suggested [4, 5].

The diaphragm is the main inspiratory muscle and, compared with peripheral muscles, appears to be more affected by critical illness and excessive respiratory drive leading to over-exertion [6]. A *post-mortem* study by Shi *et al*. (2020) in COVID-19 intensive care unit (ICU) patients reported angiotensin-converting enzyme 2 (ACE-2) expression in the diaphragm, providing an entry point for SARS-CoV-2 to infect diaphragm myofibres [7, 8]. It also found increased expression of genes involved in fibrosis and histological evidence for the development of fibrosis in the diaphragm of COVID-19 ICU patients, distinctly different from that of control ICU patients. We hypothesise that diaphragm function has a long recovery time after COVID-19 and may play a role in post-COVID-19 syndrome.

Thoracic ultrasound could provide a more feasible method to evaluate diaphragm function in recovering COVID-19 patients compared to phrenic nerve conduction studies or needle electromyogram (EMG), and can be reliably repeated over time [9]. Thoracic ultrasound can demonstrate atrophy and impaired contractility of the diaphragm [10–12]. Normal values for diaphragm thickening fraction (TF) and muscle thickness have been published and can be used for group comparison [13, 14]. Several studies conclude that thoracic ultrasound is superior to fluoroscopy for the diagnosis of diaphragm dysfunction [15, 16]. The aim of this study was to follow diaphragm function during hospitalisation and further recovery over 1 year in patients with COVID-19.

## Methods

### Study design

This was a prospective single-centre cohort study of COVID-19 patients who had been admitted to the nursing ward at Isala hospital, Zwolle, the Netherlands. All consecutive patients fulfilling inclusion criteria but not exclusion criteria were enrolled January and March 2021, except those admitted for <24 h. Eligible patients were ≥18 years of age with COVID-19 pneumonitis, confirmed by PCR test for SARS-CoV-2 and compatible chest imaging, who had been admitted to hospital primarily due to COVID-19 with hypoxaemia. We excluded the following patients: 1) those with pre-existing diseases of the diaphragm or neuromuscular disease; 2) those in whom the anticipated sonographic diaphragm measurements were not possible (mechanical ventilation, inability to follow vocal instructions); 3) those not able or unwilling to give written informed consent; and 4) those living outside the hospital region. All patients were treated according to the local treatment protocol. Permission for this study was obtained from the Medical Research and Ethics Committee Isala Clinics (number: 210120), and all patients gave written informed consent.

### Primary outcome: diaphragm assessment

**FIGURE 1:**
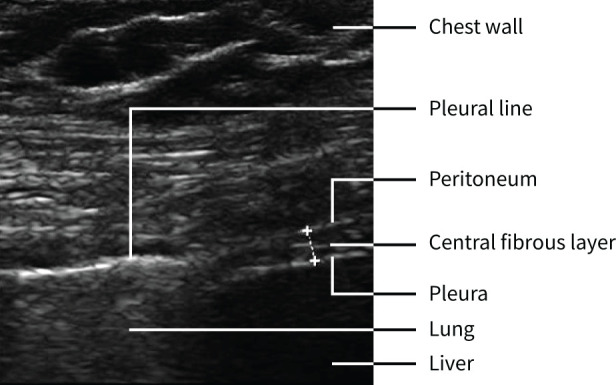
Ultrasound image of the diaphragm at the zone of apposition.

Diaphragm function was assessed within 24 h of enrolment and repeated after 7 days, or at hospital discharge, whichever came first, and 3 and 12 months after enrolment. The diaphragm was measured using the Sparq Ultrasound System (Philips Healthcare, Andover, MA, USA) with a 12 MHz linear array transducer. Diaphragm thickness was measured with the patient in supine position and the ultrasound probe perpendicular to the diaphragm between the intercostal approaches at the right mid-axillary line around the 10th intercostal space, the zone of apposition (figure 1) [13, 17–19]. The same location was used for sonographic follow-up.

The primary outcome parameter was diaphragm TF as a quantitative measure of diaphragm strength. TF is defined as a fraction of diaphragm thickness at the end of maximal inspiration (TDinsp) and at the end of maximal expiration (TDexp): TF=(T_end-inspiration _− T_end-expiration_)/T_end-expiration_ [14, 20]. The lower limit of normal (LLN) of TF is defined as 0.20 mm and of TDexp 0.15 mm [18]. Measurements of the diaphragm function were performed in duplicate by two investigators with training and experience in ultrasound of the diaphragm. The measurement with the optimal inspiration and expiration was chosen. Afterwards, a second observer repeated the measurements, blinded to the outcome of the first observer. In case of discrepancy, consensus was reached by discussion between the two observers.

### Secondary outcomes

Secondary outcomes were rate of dyspnoea, peripheral muscle strength and quality of life directly after diaphragm assessment during all moments of follow-up. The rate of dyspnoea was assessed by using a 0–100 mm horizontal visual analogue scale (VAS) for breathlessness, with 0 signifying no breathlessness and 100 signifying the worst possible intensity of breathlessness [21]. Peripheral muscle strength was measured by using handheld dynamometry using the Jamar Hydraulic Hand Dynamometer. Force of strength was measured in kilogrammes. Measurements were done with both hands, and the mean of both measurements was used. Overall quality of life was measured by EuroQol-5-Dimension Level (EQ-5D-5L) [22]. The EQ-5D-5L assesses health in five dimensions (mobility, self-care, usual activities, pain/discomfort, anxiety/depression), each of which has five levels of response (no problems, slight problems, moderate problems, severe problems, extreme problems/unable to). The EQ-5D-5L also includes a vertical VAS (EQ-VAS), with participants asked to mark how their health is today on a scale of 0–100. A higher number reflects a better quality of life for both the EQ-5D-5L and the EQ-VAS. The questionnaires were presented last to the patients, to limit possible influence on the sonographer's interpretations. All results of the different tests were withheld from the participant during the course of the study.

### Statistical analysis

Statistical analyses were performed using SPSS statistics 27.0 software. Categorical data were presented as n (%), and continuous data were presented as mean±sd or median (range), depending on the distribution. For analyses of the primary end-point, diaphragm function (TF), linear mixed model analysis for repeated TF measurements was performed, using the time point of measurement as a fixed effect. Bonferroni-adjustment was applied. Akaike's information criterion was used to select the covariance structure. Other data were analysed using paired sample t-test, Wilcoxon signed rank test or Mann–Whitney U-test depending on data distribution and paired or unpaired data. p-values <0.05 were considered significant.

Correlations were tested using Pearson or Spearman's correlation coefficient, depending on data distribution. Interrater reliability was analysed by using the intraclass correlation coefficient (ICC) with a two-way mixed effects model and an absolute agreement definition for single measurements: values <0.5, between 0.5–0.75, between 0.75–0.9 and >0.90 are indicative of poor, moderate, good and excellent reliability, respectively, all based on 95% confidence interval of the ICC estimate [23].

### Explorative sample size calculation

No formal sample size calculation was done since this was an explorative study on the novel topic of diaphragm recovery after COVID-19 infection. An explorative sample size calculation was based on estimating the mean diaphragm TF. The standard deviation was set at 0.5 based on literature [13], and the two-sided 95% confidence interval width was set at 0.5, which results in a minimum sample size of n=18.

## Results

### Patients

**FIGURE 2:**
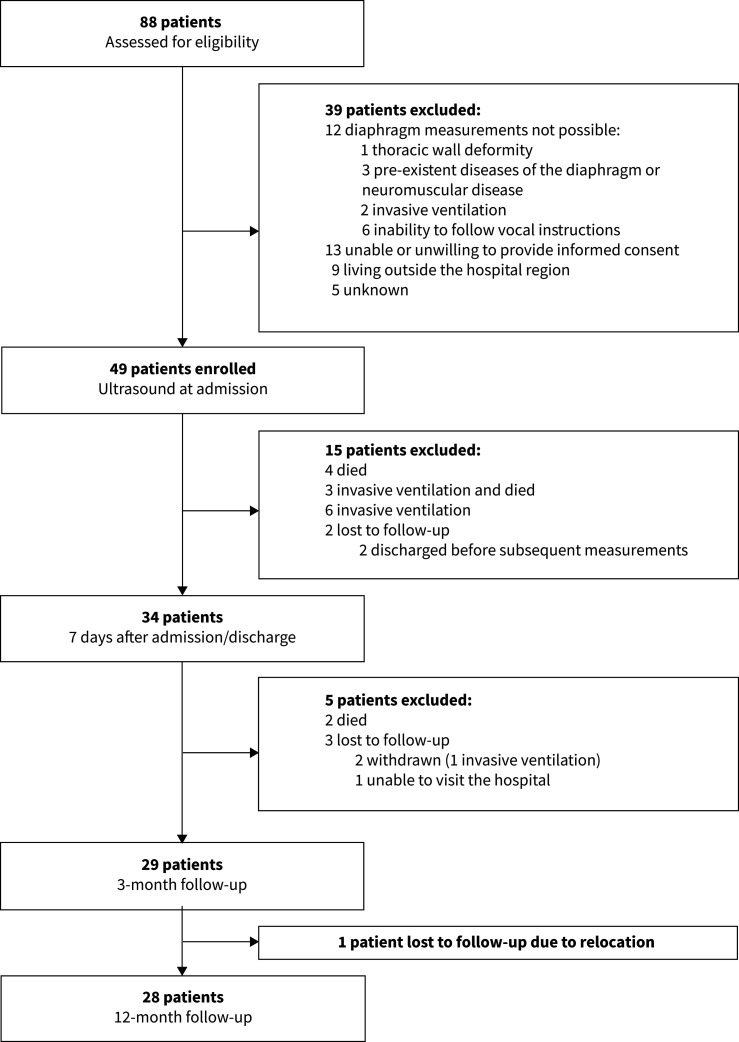
Consort flow diagram.

Between 23 January and 30 March 2021, 49 patients with COVID-19 pneumonitis were enrolled in the study shortly after admission. The recruitment and loss to follow-up are depicted in a Consort diagram (figure 2). None of the patients was diagnosed with pulmonary lung embolism, active heart failure or a secondary bacterial infection during admission. At admission median inspiratory oxygen fraction was 0.33 (range 0.21–0.85). Baseline characteristics are depicted in table 1. 34 patients were assessed by ultrasound 7 days after admission, 29 patients after 3 months of follow-up, and 28 patients after 12 months of follow-up; the reduction in patient population over time did not lead to a clear selection bias (supplementary table S1).

**TABLE 1:** Patient characteristics

**Patients n**	49
**Age years**	62 (37–85)
**Female**	12 (25)
**BMI kg·m^−2^**	28 (21–48)
**Use of steroids during admission**	49 (100)
**Smoking**	
Never	19 (39)
Current	1 (2)
Former	29 (59)
**Comorbidities**	
Diabetes mellitus	9 (18)
Hypertension	23 (47)
Cardiovascular	5 (10)
Chronic lung disease (asthma/COPD)	7 (14)
Malignancy	2 (4)
**Days of pre-hospital illness**	8 (1–28)
**Days to measuring point**	9 (3–29)
**Duration of hospitalisation days**	7 (2–54)
**Clinical characteristics on admission**	
*P*_aCO_2__ kPa	4.2 (2.8–6.0)
*P*_aO_2__ kPa	7.0 (5.3–14.3)
Respiratory rate breaths·min^−1^	23 (14–42)
*P*_aO_2__/*F*_IO_2__ ratio mmHg	235 (125–289)
**Maximal received supplemental oxygen during hospitalisation**	
Low-flow nasal cannula	21 (43)
Venturi mask (*F*_IO_2__ 0.40–0.60)	4 (8)
Non-rebreather mask	13 (27)
High-flow nasal oxygen	1 (2)
Invasive ventilation	10 (20)

Data are presented as n (%) or median (range) unless indicated otherwise. BMI: body mass index; *P*_aCO_2__: arterial carbon dioxide tension; *P*_aO_2__: arterial oxygen tension; *F*_IO_2__: inspiratory oxygen fraction.

### Primary outcome: diaphragm assessment

**FIGURE 3:**
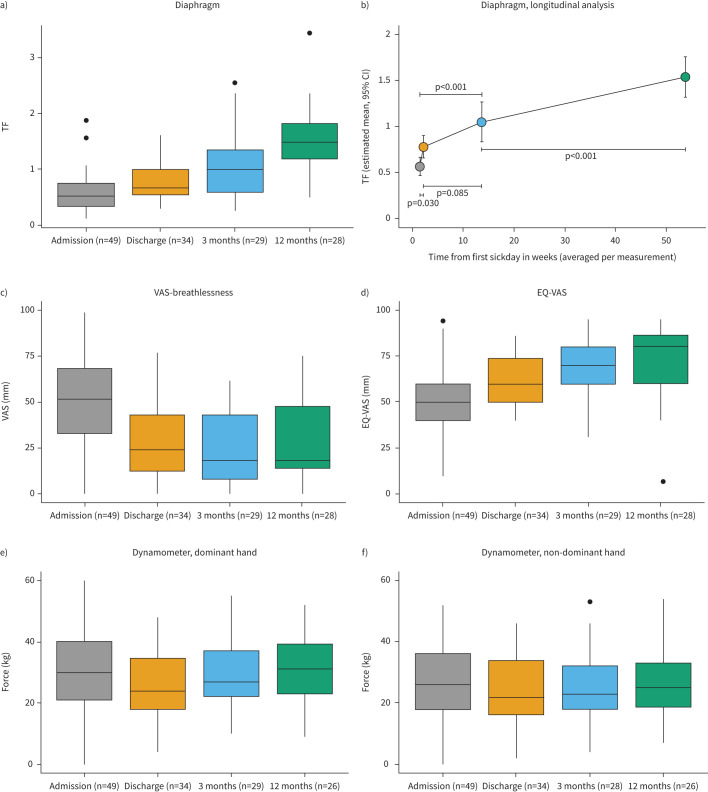
a) Median thickening fraction (TF) and minimum–maximum range at admission, at discharge or 7 days after admission, 3 months after admission and 12 months after admission. b) TF since first sick day in weeks. Data are estimated means with 95% confidence intervals, obtained from linear mixed modelling. c) Median visual analogue scale (VAS)-breathlessness at admission, day of discharge or 7 days after admission, 3 months after admission and 12 months after admission. d) Median EQ-VAS at admission, day of discharge or 7 days after admission, 3 months after admission and 12 months after admission. e) Median measurements of hand dynamometry dominant hand at admission, day of discharge or 7 days after admission, 3 months after admission and 12 months after admission. f) Median measurements of hand dynamometry non-dominant hand at admission, day of discharge or 7 days after admission, 3 months after admission and 12 months after admission.

Estimated means of diaphragm TF were 0.56 (95% CI 0.46–0.66) on the day of admission, 0.78 (95% CI 0.65–0.89) on the day of discharge/7 days after admission, 1.06 (95% CI 0.83–1.26) 3 months after admission and 1.54 (95% CI 1.31–1.76) 12 months after admission (figure 3). TF measurements improved from admission to discharge (95% CI −0.41– −0.014, p= 0.030), discharge *versus* 3-month follow-up (95% CI −0.56–0.02, p=0.085), admission *versus* 3-month follow-up (95% CI −1.06– −0.46, p<0.001) and admission *versus* 12-month follow-up (95% CI −0.72– −0.25, p<0.001) (figure 3, supplementary figure S1). Case-by-case analysis showed an improvement of TF in 26 (93%) patients from admission to 12-month follow-up; and a decrease in two (7%) patients. Of these two patients, one (−15%) showed improvement between 7 days after admission and 12-month follow-up (+96%). Lower TF on admission was associated with higher change in TF from admission to 12 months of follow-up (supplementary figure S2). At admission, four patients had a TDexp <0.15 cm and two patients had a TF <0.20, signifying that six out of 49 (12%) had abnormally low values at admission. During follow-up at 3 and 12 months none of the participants had a TF below the LLN, and six patients (21%) and three patients (11%) had an abnormal TDexp, respectively. No significant difference in TF was found between men and women (p=0.264, p=0.627, p=0.291, p=0.676, respectively at the four respective measurement time points). The ICC [23] for interrater reliability for TF for all measurements together over time was 0.905 (lower bound 0.869, upper bound 0.931).

### Secondary outcomes

Median VAS breathlessness scores showed a significant improvement between admission and discharge or 7 days after admission (p=0.001), as well as between admission and 3-month follow-up (p<0.001) and between admission and 12-month follow-up (p=0.013). No significant improvement was found between discharge and 3 months of follow-up (p=0.270), and 3 and 12 months of follow-up (p=0.477) (figure 3, supplementary table S2). No correlation was found between TF and VAS breathlessness. Mean handheld dynamometry showed significant improvement between 3-month follow-up and 12-month follow-up (p=0.004) and between discharge and 12-month follow-up (p=0.013) (figure 3, supplementary table S2).

EQ-5D-5L scores improved between baseline and 12 months for the dimensions of mobility, self-care and usual activities (p=0.020, p<0.001, p<0.001, respectively), based on 28 patients who completed follow-up. Anxiety/depression and pain/discomfort showed no change between baseline and 12 months (p=0.816, p=0.308, respectively). Self-rated health scores measured as EQ-VAS improved significantly during recovery (p<0.001) (figure 3, supplementary table S3).

## Discussion

We confirmed the presence of diaphragm dysfunction during hospitalisation for COVID-19, partially by showing abnormal values in hospital, and certainly by showing marked improvements up to 1 year. This makes our results compatible with a contribution of diaphragm dysfunction to complaints of long COVID-19 at least at 3 months.

Reports of long-lasting COVID-19 disease symptoms, so-called long COVID, are rapidly rising. A recent systematic review suggested an estimated prevalence of 0.34 in non-hospitalised patients and 0.54 in hospitalised patients [24]. The exact reason why some patients experience long-term symptoms after COVID-19 is uncertain. Patients suffering from long COVID-19 report a wide range of symptoms, but most reported symptoms are fatigue and dyspnoea [25, 26]. Underlying mechanisms which are suggested among others are long-term biochemical and inflammatory response pathways or hypoxaemia secondary to the destruction of capillaries [27, 28]. Thus far, the cause of dyspnoea has not been attributed to one mechanism, and many contributing factors have been suggested, such as altered diffusion capacity and restrictive pattern as well as obstructive pattern [29]. Another proposed mechanism is diaphragm dysfunction. This study confirms that at admission to hospital diaphragm dysfunction is present, though the number of patients with decreases in TF that can be labelled as clearly clinically abnormal is small in our study. The improvement of TF in the first 7 days of recovery in hospital, however, suggests that the values are below normal values for that patient, and given the further improvement up to 1 year, their individual normal values are on average not reached for at least a year.

Handheld dynamometry measures the isometric force of the hand. It could be a good predictor of the overall peripheral muscle strength and has already been used in several studies about lung rehabilitation [30]. Handheld dynamometry only slightly improved over time in our study, with most improvement between 3 and 12 months of follow-up, suggesting the diaphragm may be more affected than mean peripheral muscle strength in COVID-19. Several studies have assessed VAS breathlessness to quantify the severity of breathlessness, with the highest score during admission [31]. Interestingly, in our study, while diaphragm function was still recovering until 12 months of follow-up, VAS breathlessness stopped showing significant improvement. Our study suggests that recovering COVID-19 patients mention a poor quality of life, which is already demonstrated by a recent meta-analysis [22]. However, only a limited number of patients mentioned anxiety or depressive feelings, which is in contrast to the suggestion that COVID-19 patients could develop a post-traumatic stress disorder.

Theoretical mechanisms of diaphragm dysfunction in COVID-19 patients are possibly multifold and could include critical illness myopathy, ventilator-induced diaphragm dysfunction, iatrogenic phrenic nerve injury, post-infectious inflammatory neuropathy of the phrenic nerve and possibly direct neuromuscular involvement of SARS-CoV-2 [6, 8, 32–34]. Diaphragm injury has been shown in non-ventilated patients after excessive respiratory drive, manifesting as a loss in force-generating capacity and sarcomere disruption on histology [35]. Potential factors inducing excessive respiratory drive include hypoxaemia, hypercapnia, stimulation of lung and chest wall receptors, cortical stimuli and brain stem inflammation [36–39]. Understanding the level of neuromuscular involvement, including that of the diaphragm, in COVID-19 and chronic disabilities is an active area of research. A *post mortem* study in 26 patients reported ACE-2 expression in the diaphragm of COVID-19 ICU and control ICU patients providing an entry point for COVID-19 to infect diaphragm myofibres, which suggested a possible infectious involvement of the diaphragm [7, 8].

Ultrasound may be a promising and well-tolerated tool for longitudinal assessment in patients with acute or chronic respiratory symptoms, especially post-infection, and is a noninvasive procedure with widespread availability. Unfortunately, it is also a technique known for interobserver variability. To overcome this, we performed measurements in duplicate by two independent observers, and our study interrater reliability was excellent [23]. With appropriate protocols and training, ultrasound can be performed by radiology services or even as a hands-on investigation during planned hospital visits. This study followed an algorithm including ultrasound for suspected diaphragm dysfunction which has already been published [17, 20].

Another strength of our study is the long follow-up time of 1 year. Our study also has several limitations. Not all patients could be followed until 1-year follow-up, not least because of death or admission to the ICU. However, selection bias introduced this way seems to be limited at least for the parameters assessed (supplementary table S1e). Furthermore, a relatively small number of women were included. Recent studies suggest distinct values for the LLN of end-expiratory diaphragm thickness in men and women; however TF was similar regardless of sex [40]. Several studies regarding normal values have been published, but large validation studies are lacking. It would have been helpful to have pre-COVID-19 diaphragm function data to determine whether the 1-year values had normalised, but these data were not available due to the study design. Moreover, appropriate patient effort is needed for accurate measurement of TF, which may have been insufficient in acutely ill patients [13]. Finally, combined diaphragm function measurements using trans-diaphragmatic pressure measurement, EMG or both would have been interesting. However, this information was not available in the clinical setting due to the invasive setting of these procedures. Overall, we consider this explorative study provoking, and encourage replication and extension.

In conclusion, impaired diaphragm function during hospitalisation for COVID-19 improved during long-term follow-up, even between 3 and 12 months of follow-up, suggesting a long recovery time of the diaphragm. Diaphragm ultrasound may be a valuable modality in the screening and follow-up of (post-)COVID-19 patients for diaphragm dysfunction.

## Supplementary material

10.1183/23120541.00623-2022.Supp1 **Please note:** supplementary material is not edited by the Editorial Office, and is uploaded as it has been supplied by the author.
Supplementary material 00623-2022.supplement
