# Supplementary material for: Sonographic follow-up of diaphragm function in COVID-19: an exploratory study
Source: ERJ Open Res. 2023 May 2;9(3):00623-2022. doi: 10.1183/23120541.00623-2022 (PMC10152246; doi:10.1183/23120541.00623-2022)

Supplemental File

Table S1. Patient characteristics over time.

**Characteristic**	**Admission**	**Discharge**	**3 months**	**12 months**
Number of patients	49	34	29	28
Median age in years (range)	62 (37-85)	61.5 (43-85)	61 (43-85)	61,5 (43-85)
Female (%)	12 (25)	12 (35)	10 (35)	9 (32)
Median BMI kg/m2 (range)	28 (21 - 48)	27 (21-48)	27 (21-48)	27 (21-38)
Use of steroids during admission (%)	49 (100)	34 (100)	29 (100)	28 (100)
Smoking<br>- Never (%)<br>- Current (%)<br>- Former (%)	 19 (39)<br>1 (2)<br>29 (59)	 15 (44)<br>1 (3)<br>18 (53)	 14 (48)<br>1 (3)<br>14 (48)	 13 (46)<br>1 (4)<br>14 (50)
Comorbidities<br>- Diabetes mellitus (%)<br>- Hypertension (%)<br>- Cardio-vascular (%)<br>- Chronic lung disease (asthma/COPD) (%)<br>- Malignancy (%)	 9 (18)<br>23 (47)<br>5 (10)<br>7 (14)<br>2 (4)	 8 (24)<br>17 (50)<br>5 (15)<br>6 (18)<br>2 (6)	 6 (21)<br>15 (52)<br>3 (9)<br>4 (14)<br>1 (3)	 6 (21)<br>15 (54)<br>3 (11)<br>4 (14)<br>1 (4)
Days of prehospital illness (median, range)	8 (1-28)	8 (1-28)	9 (1-28)	9,5(1-28)
Days to measuring point (median, range)	9 (3-29)	13,5 (8 - 32)	94 (77-117)	377 (339 - 434)
Duration of hospitalization in days (median, range)	7 (2-54)	6 (2 - 37)	5 (2 - 37)	5 (2 - 37)
Clinical characteristics on admission<br>- PaCO2 (kPa) (median, range)<br>- PaO2 (kPa) (median, range)<br>- Respiratory Rate (breaths/min) (median, range)<br>- PaO2/FiO2 ratio (mmHg) (median, range)	 4,2 (2,8 - 6,0)<br>7,0 (7,0 - 14,3)<br>23 (14 - 42)<br>235 (125 - 289)	 4,3 (2,8 - 6,0)<br>7,0 (5,6 - 9,3)<br>22 (14 - 33)<br>246 (145 - 289)	 4,3 (2,8 - 5,9)<br>7,2 (6,3 - 9,1)<br>22 (14 - 33)<br>246 (145 - 289)	 4,3 (2,8 - 5,9)<br>7,2 (6,3 - 9,1)<br>22 (14 - 33)<br>246 (145 - 289)
Maximal received supplemental oxygen during hospitalization (%)<br>- Low-flow nasal cannula	 21 (43)	 19 (56)	 17 (59)	 17 (61)

- Venturi mask (FiO2 0.40-0.60)	4 (8)	4 (12)	4 (14)	4 (14)
- Non-rebreather mask	13 (27)	9 (26)	8 (28)	7 (25)
- High-flow nasal oxygen	1 (2)	1 (3)	0 (0)	0 (0)
- Invasive ventilation	10 (20)	1 (3)	0 (0)	0 (0)

Table S2. Secondary outcomes.

**Secondary outcome**	**Admission vs. discharge**	**Discharge vs. 3 month follow-up**	**3 months follow-up vs. 12 months follow-up**	**Admission vs. 3 months follow-up**	**Admission vs. 12 months follow-up**
**VAS breathlessness (median)**	52 (0-99) vs. 24 (0-77) **p=0.001**	24 (0-77) vs. 18 (0-62) p=0.270	18 (0-62) vs. 18 (0-75) p=0.477	52 (0-99) vs. 18 (0-62) **P<0.001**	52 (0-99) vs. 18 (0-75) **p=0.013**
**Hand held dynamometry (mean in kg)**	28 (±13) vs. 25 (±11) p=0.736	25 (±11) vs. 27 (±12) p=0.508	27 (±12) vs. 30 (±13) **p=0.004**	28 (±13) vs. 27 (±12) p=0.489	28 (±13) vs. 30 (±13) **p=0.025**

Table S3. EQ-5D-5L results.

Dimension	Admission n (%)	Discharge n (%)	3 months follow-up n (%)	12 months follow-up n (%)	Admission vs. Discharge *P* value	Admission vs. 3 months follow-up *P* value	Admission vs. 12 months follow-up *P* value
**Mobility**							
No problems	8(16,7)	12 (35,3)	15(51,7)	16(57,1)	0,120	0,005	0,020
Slight problems	18(37,5)	10(29,4)	8(27,6)	5(17,9)			
Moderate problems	13(27,1)	5(14,7)	5(17,2)	3(10,7)			
Severe problems	7(14,6)	2(14,7)	1(3,4)	4(14,3)			
Unable	2(4,2)	5(14,7)	0(0)	0(0)			
**Self-care**							
No problems	12(24,5)	12(35,3)	26(89,7)	27(96,4)	0,102	<0,001	<0.001
Slight problems	11(46,9)	12(35,3)	3(10,3)	1(3,6)			
Moderate problems	16(32,7)	8(23,5)	0(0)	0(0)			
Severe problems	6(12,2)	0(0)	0(0)	0(0)			
Unable	4(8,2)	2(5,9)	0(0)	0(0)			
**Usual activities**							
No problems	8(16,3)	7(20,6)	12(41,4)	13(46,4)	0,024	<0,001	<0,001
Slight problems	4(24,5)	6(17,6)	6(20,7)	7(25,0)			
Moderate problems	9(18,4)	10(29,4)	11(37,9)	6(21,4)			
Severe problems	6(12,2)	1(2,9)	0(0)	2(7,1)			
Unable	22(44,9)	10(29,4)	0(0)	0(0)			
**Pain/discomfort**							
No problems	21(42,9)	19(55,9)	17(58,6)	10(35,7)	0,005	0,030	0,816

Slight problems	9(18,4)	10(29,4)	6(20,7)	10(35,7)			
Moderate problems	17(34,7)	5(14,7)	6(20,7)	7(25,0)			
Severe problems	2(4,1)	0(0)	0(0)	1(3,6)			
Extreme problems	0(0)	0(0)	0(0)	0(0)			
**Anxiety/depression**							
No problems	30(61,2)	25(73,5)	23(79,3)	21(75,0)	0,193	0,088	0,308
Slight problems	7(14,3)	6(17,6)	5(17,2)	5(17,9)			
Moderate problems	8(16,3)	1(2,9)	1(3,4)	2(7,1)			
Severe problems	2(4,1)	1(2,9)	0(0)	0(0)			
Extreme	1(2,0)	1(2,9)	0(0)	0(0)			
**EQ VAS**	Mean(SD)	Mean(SD)	Mean(SD)	Mean(SD)			
	49,0(17,7)	61,5(13,5)	70,9(13,3)	72,5(19,8)	0.001	<0,001	<0,001

Figure S1. Linear mixed model analysis of estimated means with changes in TF from baseline (grey) in percentage at discharge or 7 days after admission (yellow), three months after admission (blue) and twelve months after admission (green).

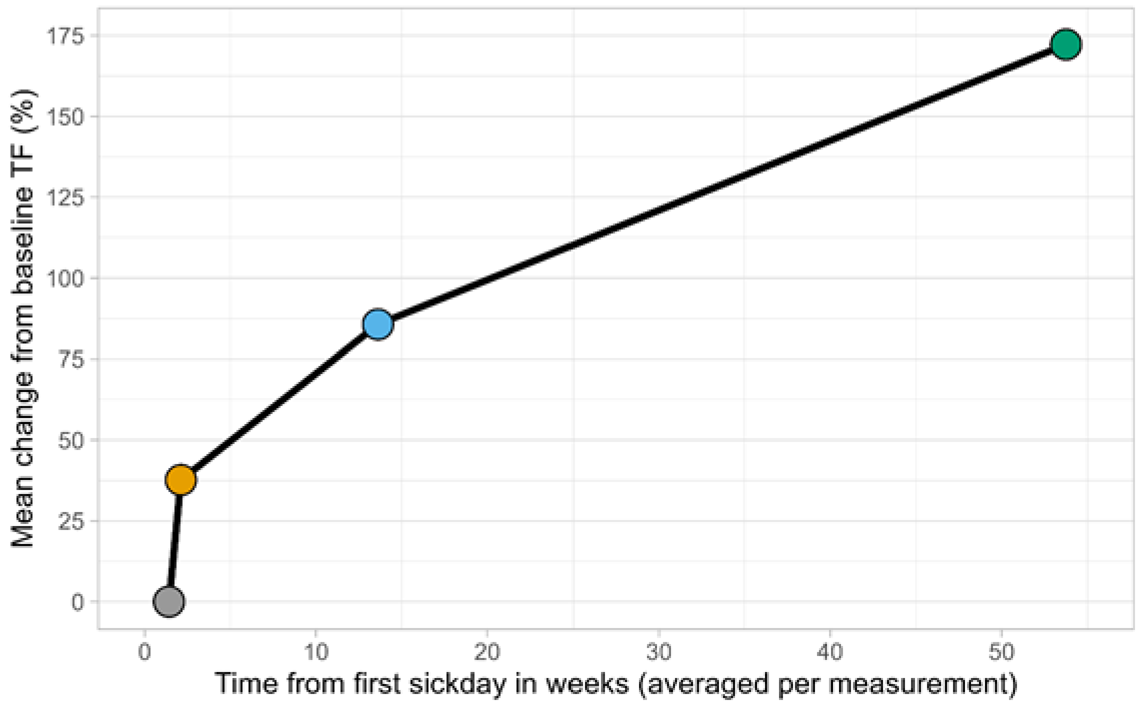

Figure S2. Association between baseline TF (on admission) and change in TF from admission to twelve months of follow-up with curve fitting based on geometric smoothing method.

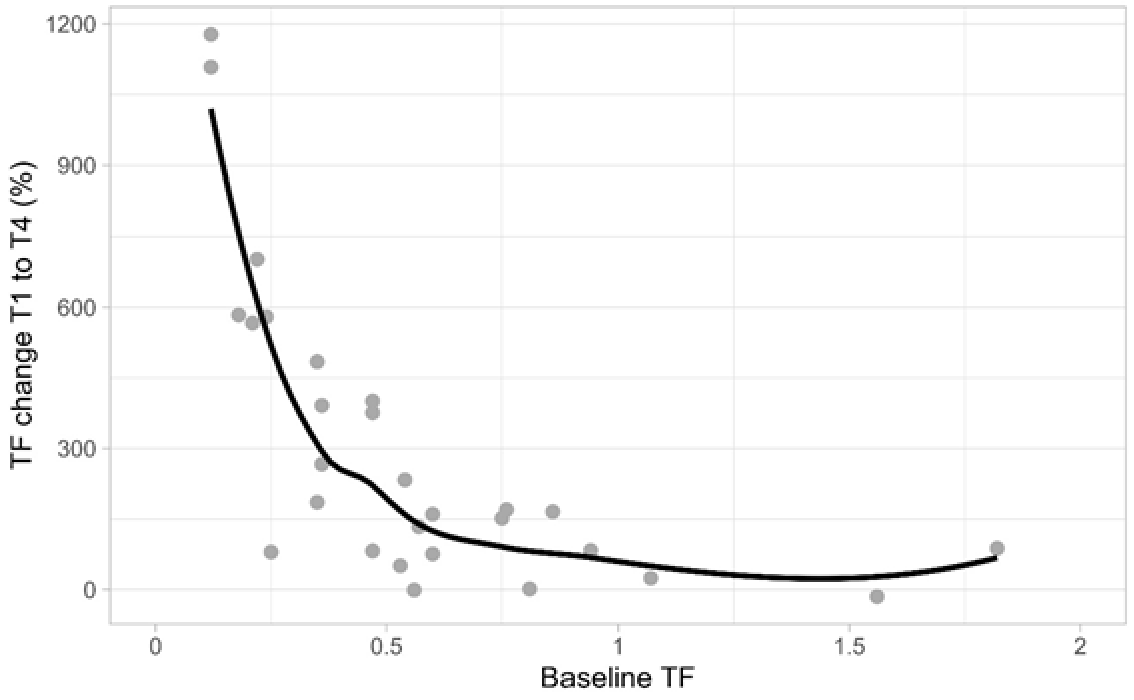

Supplement: Supplementary file 1 [file 00623-2022.supplement.pdf]
